# Inflammatory pathway communication with skeletal muscle—Does aging play a role? A topical review of the current evidence

**DOI:** 10.14814/phy2.16098

**Published:** 2024-06-14

**Authors:** Stephen M. Cornish, Dean M. Cordingley

**Affiliations:** ^1^ Faculty of Kinesiology and Recreation Management University of Manitoba Winnipeg Manitoba Canada; ^2^ Applied Health Sciences University of Manitoba Winnipeg Manitoba Canada; ^3^ Centre for Aging University of Manitoba Winnipeg Manitoba Canada; ^4^ Pan Am Clinic Foundation Winnipeg Manitoba Canada

**Keywords:** aging, exercise, inflammation, skeletal muscle

## Abstract

Skeletal muscle plays an integral role in locomotion, but also as part of the integrative physiological system. Recent progress has identified crosstalk between skeletal muscle and various physiological systems, including the immune system. Both the musculoskeletal and immune systems are impacted by aging. Increased age is associated with decreased muscle mass and function, while the immune system undergoes “inflammaging” and immunosenescence. Exercise is identified as a preventative medicine that can mitigate loss of function for both systems. This review summarizes: (1) the inflammatory pathways active in skeletal muscle; and (2) the inflammatory and skeletal muscle response to unaccustomed exercise in younger and older adults. Compared to younger adults, it appears older individuals have a muted pro‐inflammatory response and elevated anti‐inflammatory response to exercise. This important difference could contribute to decreased regeneration and recovery following unaccustomed exercise in older adults, as well as in chronic disease. The current research provides specific information on the role inflammation plays in altering skeletal muscle form and function, and adaptation to exercise; however, the pursuit of more knowledge in this area will delineate specific interventions that may enhance skeletal muscle recovery and promote resiliency in this tissue particularly with aging.

## 1 INTRODUCTION

In humans, skeletal muscle is a dynamic tissue that allows for locomotion to carry out activities of daily living, participates in physical activity or exercise, and is highly relevant to maintaining functional independence even into older age. More recently, the endocrine role of skeletal muscle in communicating with other physiological systems has been somewhat elucidated (Chow et al., 2022; Severinsen & Pedersen, 2020). The immune system is a complex physiological system that provides defense against foreign organisms that may bring illness or disease. The crosstalk between skeletal muscle and the immune system, particularly the innate immune system's inflammatory response, requires further exploration to completely understand the mechanisms underlying the communication between these two systems, especially during the aging process (Tidball, 2017).

With aging, there are changes to multiple physiological systems, and the immune system and skeletal muscle are without exception (Fountain et al., 2023; Liang et al., 2022; Müller et al., 2019; Pedersen et al., 2004; Ross et al., 2019). The immune system undergoes immunosenescence, which increases the risk for infectious disease, and “inflamm‐aging,” the chronic low‐grade inflammatory process that increases the risk for several non‐communicable diseases that may affect many physiological systems including the musculoskeletal system (Liang et al., 2022). With an aging musculoskeletal system, there is the loss of skeletal muscle mass, function, and potentially its endocrine ability (Fountain et al., 2023; Pedersen et al., 2004). This in turn reduces functional ability, independent living, and increases the risk of diseases associated with poor skeletal muscle endocrine capability. The term sarcopenia has been used to define the loss of skeletal muscle mass and functional ability with age; however, research indicates that chronic low‐grade inflammation found in aging is potentially one of the causes responsible for altering skeletal muscle form and function by modifying the inflammatory response to exercise or reducing the anabolic capacity of this tissue (Aragon et al., 2023; Fountain et al., 2023; Peake et al., 2010).

While changes to both these systems occur with aging, exercise training may preserve or maintain function and reduce the negative consequences of aging on these two physiological systems (Morawin et al., 2023; Peake et al., 2010). In fact, exercise training has been singled out as a first line of preventative medicine for many non‐communicable diseases and aids in the treatment of disease if it becomes established (Tao et al., 2023). Exercise training involves skeletal muscle actions (either concentric, eccentric, or isometric or a combination of these) and is typically found in the form of aerobic exercise or resistance exercise training. Aerobic exercise skeletal muscle actions include continuous muscle actions of longer duration in a repetitive fashion done to enhance the functioning of the cardiovascular system and the ability of the body to deliver oxygen to the working skeletal musculature via enhanced oxidative phosphorylation. Resistance exercise involves skeletal muscle actions that are applied against one's own body mass (i.e., body‐weight exercises) or against an external resistance that can be done using free weights, machines, or other implements to enhance skeletal muscle function/strength and maintain or enhance skeletal muscle size. With consistent exercise training, both aerobic and resistance exercise have localized and multi‐system physiological benefits for the participant (Abou Sawan et al., 2023; Tao et al., 2023).

In our review, there are three main parts that will focus on the crosstalk between skeletal muscle and the inflammatory response. These include (1) an overview of the main inflammatory pathways of the immune system that are active in skeletal muscle; (2) healthy young adults' skeletal muscle and inflammatory response to unaccustomed exercise; and (3) older adults' skeletal muscle and inflammatory response to unaccustomed exercise. The review will then provide a conclusion and future research recommendations for this area of study.

## 2 INFLAMMATORY RESPONSE PATHWAYS IN SKELETAL MUSCLE

It is important to define terminology clearly regarding what is meant by terms associated with the inflammatory reaction in skeletal muscle. The terms regeneration, repair, and adaptation are sometimes used synonymously but do have different meanings. Grounds (2014) discussed these processes in their manuscript. Here, the definition of skeletal muscle regeneration is that it is “used for events that follow myofiber necrosis, to result in myogenesis and new muscle formation.” The author goes on to state: “regeneration is not restoration of muscle mass by hypertrophy after atrophy and other forms of damage to muscle tissue components.” (Grounds, 2014). Repair of skeletal muscle tissue is considered a part of the regenerative process (Laumonier & Menetrey, 2016). However, adaptation is focused more on exercise or drug induced hypertrophy of skeletal muscle tissue and may or may not involve the regenerative process (Grounds, 2014). Furthermore, in an excellent review on skeletal muscle regeneration, the process of phagocytosis is essential to myogenic regeneration once damage and degeneration of fibers has occurred (Anderson, 2022). The acute inflammatory reaction to injury or insult in skeletal muscle is a necessary response to ensure it goes through the designated processes which ultimately lead to resolution of the injury or damage and a regeneration of skeletal muscle structure and function. In the past, scientists hypothesized that there was some level of communication between the immune/inflammatory response and skeletal muscle damage; however, it is only recently that the level of synchronization between the two systems has been further illuminated (Tidball, 2017). Certain pro‐inflammatory cytokines (i.e., interleukin‐1β (IL‐1β), IL‐6, and tumor necrosis factor‐α (TNF‐α)) and microbial agents (bacteria, viruses, and fungi) are the main stimuli for inflammation (Chen et al., 2018). Typical intracellular transcription pathways for inflammatory response activation include Janus kinase (JAK)‐signal transducer and activator of transcription (STAT), mitogen‐activated protein kinase (MAPK), and the nuclear factor kappa‐light‐chain‐enhancer of activated B‐cell (NF‐κB) pathways (Chen et al., 2018). In the process of the inflammatory response to skeletal muscle damage, the innate cellular immune reaction responds to the pro‐inflammatory stimuli by increasing extravasation of leukocytes, in particular first neutrophils and later monocytes/macrophages, to the damaged area to initiate the inflammatory response and later to bring about resolution of the damage (Tidball, 2017; Tiidus, 2008). In relation to the previous point, neutrophils respond first to promote the inflammatory reaction, followed by M1‐phenotypic macrophages (which are predominantly pro‐inflammatory in nature and activated by T helper 1 type cytokines); this is then followed up by M2‐phentoypic macrophages (which are predominantly involved in the resolution of inflammation and activated by the T helper 2 type cytokines) (Tidball, 2017). Tidball (2017) makes it clear, there is a range of phenotypes between the M1 and M2 dichotomy of macrophages that is currently underappreciated, and some research has indicated a mix of M1 and M2 phenotypes with varying functional characteristics different from what is typically dichotomized (i.e., pro‐inflammatory for M1 and anti‐inflammatory for M2). Other cells of the adaptive immune system respond later in the process such as CD8+ T cells (cytotoxic or killer T cells) and then T regulatory cells which aid in the regeneration of skeletal muscle tissue and a cessation of the inflammatory response (Tidball, 2017). Overall, the inflammatory events initiated by the immune system to damaged or injured skeletal muscle are a highly coordinated process (see Table 1 for overview of cells leukocytes involved in the process) that involves both soluble mediators (such as cytokines) and cellular responses with various leukocytes of the immune system as mentioned above.

**TABLE 1:** Main types of immune cells involved in skeletal muscle regeneration and repair.

Cell type	Immune function	Skeletal muscle role
Neutrophil	Chemotaxis to site of injury or infection; phagocytosis; degranulation for antimicrobial function	Attracted to site of skeletal muscle injury first responding to pro‐inflammatory cytokines via chemotaxis
M1 Macrophage	Pro‐inflammatory—secrete IL‐12; bactericidal; phagocytic	Involved in initial inflammatory reaction within muscle to injury or damage.
M2 Macrophage	Anti‐inflammatory; pro‐regenerative	Involved in producing anti‐inflammatory cytokines such as IL‐10; resolving inflammation; tissue repair
CD4+ T‐Cell (Helper)	Involved in adaptive immunity; activate CD8+ T cells; crosstalk with macrophages and neutrophils	Regulates angiogenesis and myogenesis
CD8+ T‐Cell (Cytotoxic)	Cytotoxic role within the immune system; release of TNF‐α and IFN‐γ	Role in recruitment of other immune cells largely driven by the release of TNF‐α and IFN‐γ
T Regulatory Cell	Enhance ability to detect self from non‐self; promote production of TGF‐β and IL‐10 (inhibitory cytokines)	Release IL‐10 which promotes phenotypic shift of macrophages; release of amphiregulin to increase production of myogenic regulatory factors

*Note*: Adapted from Tidball (2017).

## 3 INFLAMMATORY RESPONSE IN YOUNG ADULT SKELETAL MUSCLE

**FIGURE 1:**
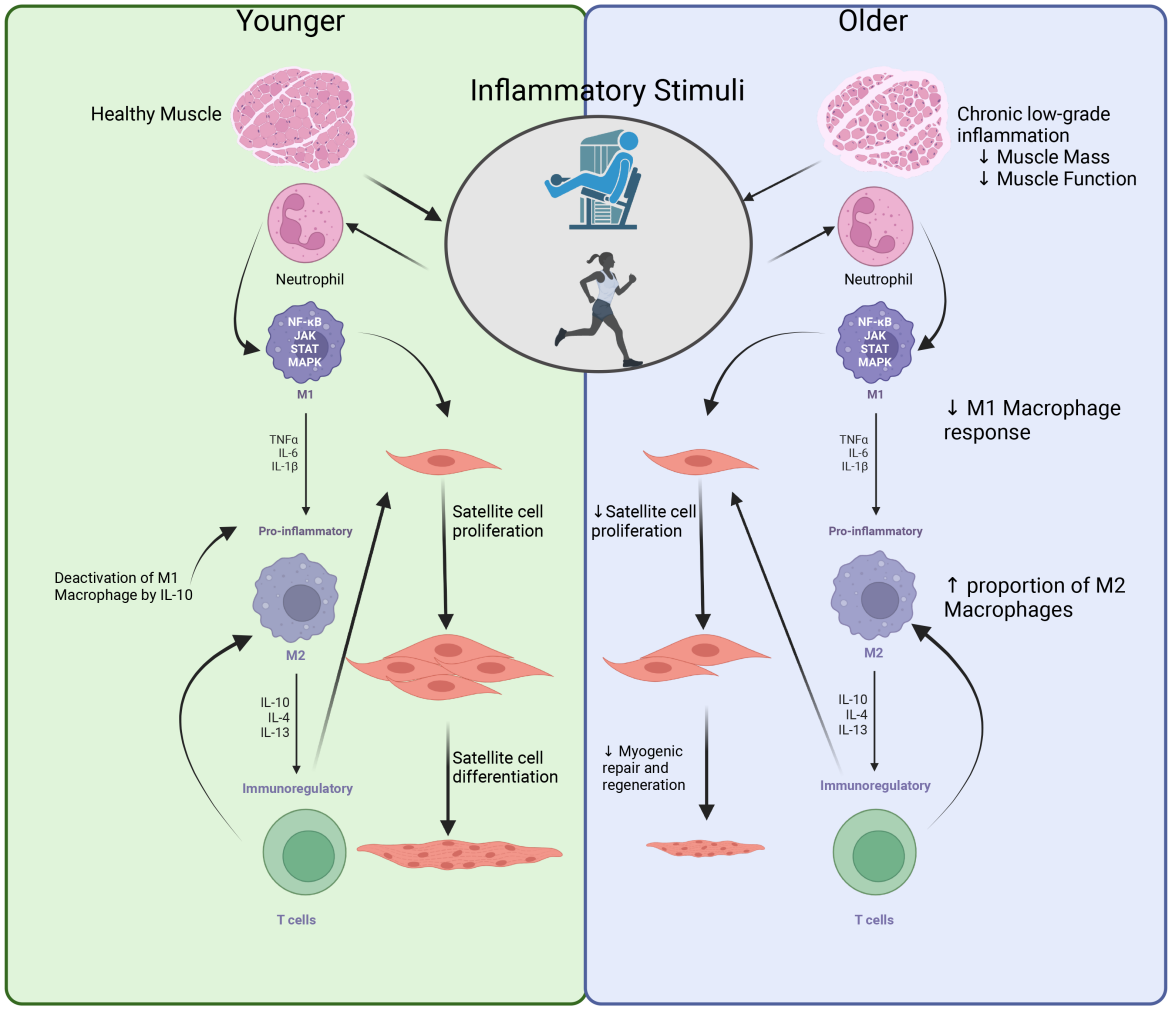
A comparison of younger healthy skeletal muscle and older chronically inflamed skeletal muscle inflammatory response to unaccustomed exercise or exercise with an eccentric contraction component to it (i.e., inflammatory stimuli). Neutrophils are recruited to the site of skeletal muscle damage first via interleukin‐6 (IL‐6) and IL‐8. This stimulates the recruitment of M1 macrophages to the skeletal muscle and activation of the various inflammatory pathways including Janus kinase (JAK)‐signal transducer and activator of transcription (STAT), mitogen‐activated protein kinase (MAPK), and the nuclear factor kappa‐light‐chain‐enhancer of activated B cells (NF‐κB). M1 macrophages (pro‐inflammatory) stimulate the proliferation of satellite cells in skeletal muscle and the inflammatory response via secretion of pro‐inflammatory cytokines. M2 macrophages (anti‐inflammatory and pro‐regenerative) then converge in the area to allow for regeneration of skeletal muscle tissue via the secretion of anti‐inflammatory cytokines. Finally, T cells (specifically T regulatory cells) are recruited to the area of regeneration and signal satellite cells to proliferate and differentiate. Older skeletal muscle that is chronically inflamed typically has less M1 macrophages recruited to the area and an increased proportion of M2 macrophages which may decrease satellite cell proliferation and differentiation and delay regeneration and repair of skeletal muscle.

As stated, the inflammatory response to skeletal muscle damage due to unaccustomed exercise is highly coordinated. Figure 1 describes the differences that exist between young and older skeletal muscle inflammatory responses that will be expanded on below. In young, healthy adults, this response ensures the eventual restoration of functional ability that may match or exceed the capability of the former skeletal muscle. With resistance type exercise, the eventual result is an enhancement of muscle protein synthesis which, if chronically upregulated, will result in skeletal muscle hypertrophy. With aerobic exercise training, there is a typical adaptation where oxygen utilization is improved due to several biological changes and enhanced capacity for oxidative phosphorylation to produce adenosine triphosphate, the main energy currency used by skeletal muscle. A recent review on the topic has delineated that certain health and/or performance effects are developed from both types of exercise training including glucose homeostasis, mobility, body composition, mitochondrial function, and cognitive function (Abou Sawan et al., 2023). Nonetheless, when unaccustomed exercise is completed, the inflammatory reaction within skeletal muscle is typical and initiates the response to the physical stress placed on the muscle.

Neutrophils are the initial leukocyte to enter skeletal muscle following muscle damaging exercise. In an elegant study design evaluating both skeletal muscle and blood‐based neutrophils following 2 h of endurance‐based exercise in young adult males, Broadbent et al. (2017) demonstrated through weighted gene coexpression network analysis that there was a strong relationship between the two subsets regarding mitochondrial related networks and acetylation (i.e., the posttranslational reversible modification of proteins). Furthermore, correlations were exhibited between skeletal muscle gene subnetworks and neutrophil, monocyte, and total leukocyte counts as well as a blood‐based cytokine (IL‐6), C‐reactive protein, and creatine kinase activity (Broadbent et al., 2017). The authors of this study concluded the information reveals important crosstalk between skeletal muscle and the innate immune system/inflammatory response to exercise and that blood‐based biomarkers, a less invasive method of assessment than skeletal muscle biopsy, may be one method to understand the inflammatory response to exercise in skeletal muscle (Broadbent et al., 2017). The results of the aforementioned study are tempered by other findings which indicate systemic concentrations of cytokines do not always correlate with local skeletal muscle tissue concentrations of cytokines thus indicating that inflammation at the target organ of interest (i.e., skeletal muscle) is not necessarily the same as what may be suggested by systemic inflammatory biomarkers (Paulsen et al., 2012). It may be that the up‐regulation of systemic cytokines in circulation are more indicative of alterations or feedback mechanisms for metabolism (Paulsen et al., 2012; Pedersen et al., 2004).

The M1 and M2 types of macrophages are essential to proper signaling to satellite cells after skeletal muscle damage to initiate and conclude the repair process. If the M1 macrophage persists for a longer period (beyond 2 days of recovery), then there can be delays in the repair process and an exacerbated inflammatory response (Shireman et al., 2007). M1 macrophages are known to interact with satellite cells by initiating their migration and proliferation (Perandini et al., 2018). Conversely, if the M2 macrophage presents too early or persists for longer, there may be an alteration and dysregulation in tissue healing as well as satellite cell control of the regenerative process (Perdiguero et al., 2011). M2 macrophages are known to commence the differentiation process of satellite cells in muscle (Perandini et al., 2018). In young, healthy adult skeletal muscle tissue, this process is highly regulated and coordinated with M1 and M2 macrophages (along with their role in satellite cell activation) playing a key role at crucial times to result in the regenerative process in skeletal muscle (Ahmadi et al., 2022).

Interestingly, it has been thought that eccentric type resistance exercise, resulting in a greater inflammatory response, would produce more skeletal muscle hypertrophy regarding the chronic adaptations observed in comparison with concentric only resistance exercise. A recent study has contradicted this by demonstrating that isokinetic dynamometer resistance exercise training completed over 12 weeks using eccentric and concentric exercise on one leg and concentric only on the opposite leg saw no differences in improvements made to cross‐sectional area, muscle volume, or muscle strength, despite more work being applied to the leg that completed the eccentric and concentric exercise bouts (Mallinson et al., 2020). The remarkable conclusion of this study is that despite early changes in skeletal muscle mRNA expression that indicates the eccentric/concentric leg training resulted in more abundance of mRNA related to cell function, this did not translate to improve skeletal muscle cross‐sectional area (Mallinson et al., 2020). This result contextualizes a possible phenomenon that may be occurring in this within limb study design. Is it possible that myokine release from the unilaterally exercised skeletal muscle was having a distal effect on the contralateral limb's musculature thus negating any differences the study found in muscle cross‐sectional area, muscle volume, and muscle strength? This is not beyond the realm of possibilities as skeletal muscle has been postulated to act in an endocrine manner via the release of myokines systemically (Chow et al., 2022). For example, Mallinson et al. (2020) found an increased IL‐6 mRNA content in the eccentric/concentric exercised leg after 24 h and 7 days of exercise. If the increased mRNA resulted in increased protein expression of this myokine, the association of this myokine with satellite cells has been demonstrated in previous literature and may be part of the satellite cell cycle (McKay et al., 2009). This, in turn, would facilitate recovery and regeneration in skeletal muscle. Conversely, other evidence suggests that in the context of higher or lower systemic hormone concentrations (growth hormone, insulin like growth factor‐1, and testosterone) after exercise, there is no significant difference in the anabolic signaling or muscle protein synthesis with either low or high anabolic hormones following acute resistance exercise (West et al., 2009). The previous study suggested the effects of putative anabolic signaling hormones following acute resistance exercise may not affect skeletal muscle growth as there was no difference in growth between the low and high hormone within study design. This study concluded the hypertrophy process was likely more intrinsic in nature versus a response to systemic circulating anabolic factors (West et al., 2009). However, further research did demonstrate a correlation between the systemic response of IL‐6 to an acute bout of resistance exercise after a resistance training program and skeletal muscle mean fiber cross‐sectional area suggesting a role for IL‐6 in the process of muscle hypertrophy (Mitchell et al., 2013). Thus, the effects of putative anabolic hormones on muscle hypertrophy may not be as pronounced as once thought and other factors, such as IL‐6, may be participating in enhancing skeletal muscle hypertrophy following acute resistance exercise; however, this requires much further investigation.

In another unique 12‐week endurance exercise training study, bookmarked by acute bouts of resistance exercise on either ends of the training, middle‐aged females had skeletal muscle biopsies that demonstrated M2 macrophage content increasing in response to the endurance exercise training but not in response to the acute bouts of resistance exercise (Walton et al., 2019). Furthermore, this study demonstrated a significant association between the exercise induced increase in the number of M2 macrophages/fiber and muscle fiber cross‐sectional area as well as satellite cells/fiber following the endurance exercise training (Walton et al., 2019). Along with the previous result, there was immunohistochemistry staining that indicated a close proximity of M2 macrophages with Pax7+ satellite cells thus potentially demonstrating communication between the two (Walton et al., 2019). Finally, this study demonstrated an increase in the gene expression of the anti‐inflammatory cytokine interleukin‐4 (IL‐4) and a decrease in the pro‐inflammatory cytokine IL‐6 with the endurance exercise training (Walton et al., 2019). Speculatively, these results point to alterations with endurance exercise training that potentially link skeletal muscle responses and satellite cell activity with M2 macrophages. Also, a decreased inflammatory environment in middle‐aged females suggests possible crosstalk between the immune system and skeletal muscle via the cytokine's myokine role.

Within the context of younger and middle‐aged adults, the information presented above demonstrates the interaction between the immune system/inflammatory response and skeletal muscle in healthy individuals. Against the backdrop of chronic low‐grade inflammation and potentially unhealthy individuals, this review will now focus on the potential differences noted between younger and older adults in the crosstalk exhibited between these two physiological systems.

## 4 INFLAMMATORY RESPONSE IN OLD ADULT SKELETAL MUSCLE

The association between increased systemic inflammatory biomarkers as well as skeletal muscle inflammatory responses and sarcopenia is apparent in the literature (Degens & Korhonen, 2012; Draganidis et al., 2021; Peake et al., 2010). It is evident that toll‐like receptor 4 (TLR4) has a pivotal role in promoting the production of pro‐inflammatory cytokines through a variety of stimuli (such as lipopolysaccharides and damage‐associated molecular patterns) via IκB kinase‐NFκB nuclear transcription in skeletal muscle (Kunz & Lanza, 2023). In older muscle, TLR4 is increased thus potentially increasing pro‐inflammatory cytokine production via this pathway which would lead toward muscle atrophy unless mitigated by an intervention such as exercise (Ducharme et al., 2022). Furthermore, the NLRP3 inflammasome, when stimulated, produces the pro‐inflammatory cytokines IL‐1β and IL‐18, which may influence skeletal muscle negatively; however, further research into the exact effect that NLRP3 has on skeletal muscle is necessary to fully elucidate how it may be influencing this tissue during aging (Kelley et al., 2019). Nonetheless, there is evidence to suggest that aging influences the development of a chronic low‐grade inflammatory state which negatively influences skeletal muscle (Draganidis et al., 2021). In the context of aged skeletal muscle, there may be a difference in how it responds to unaccustomed, or muscle damaging exercise compared to younger, healthy adult skeletal muscle especially if the aged individuals have chronic low‐grade inflammation or are in some type of diseased state that increases inflammation. Support for this comes from a study that measured various inflammatory markers in skeletal muscle biopsies taken in a rested and fasted state from the vastus lateralis muscle of the upper thigh in young and older adults (Caldow et al., 2013). Here, the authors reported the mRNA and protein content of monocyte chemoattractant protein‐1 (MCP‐1), and the protein expression of IL‐8 was higher in the older cohort compared to the younger, suggesting a more pro‐inflammatory environment in the older adults. Nevertheless, as this study was done in fasted and rested skeletal muscle, it does not demonstrate the effects of unaccustomed exercise on the crosstalk between skeletal muscle and the innate immune/inflammatory response.

Research completed on younger and older male adults before and following an acute bout of isokinetic resistance exercise and 12 weeks of resistance exercise training determined that the inflammatory response to unaccustomed exercise tended to increase with age; however, regular resistance exercise training may normalize this response (Della Gatta et al., 2014). In this study, skeletal muscle biopsies were taken, and the protein content of various pro‐inflammatory (MCP‐1, IL‐6, and IL‐8) and anti‐inflammatory (IL‐4, IL‐10, IL‐13) cytokines was measured in the muscle homogenate. The authors concluded that there is a large increase in protein content of pro‐inflammatory cytokines in skeletal muscle with minimal increases in anti‐inflammatory cytokines in response to intensive acute isokinetic resistance exercise across the ages measured cross‐sectionally (Della Gatta et al., 2014). However, contrary to their hypothesis, there were no statistically significant differences in protein content of the cytokines measured between age and training status in this small cohort (*n* = 16). In support of this finding, another study indicated that regeneration of skeletal muscle tissue, the inflammatory response, and the angiogenic response to unaccustomed eccentric plantar flexion using an isokinetic dynamometer was not significantly different between a younger and older cohort of healthy male and female adults (Buford et al., 2014). Intriguingly, the authors summarized by stating aging per se does not influence the responses measured in the healthy cohorts of younger and older humans; nonetheless, they speculated that older unhealthy adults with comorbid conditions may respond quite differently to the physical stress of unaccustomed exercise (Buford et al., 2014). Contrasting these findings, our laboratory has demonstrated some alterations in the systemic circulation of certain myokines (leukemia inhibitory factor and irisin) between younger and healthy older adult males where both these myokines were present in higher concentrations in the younger compared to the older individuals (Cordingley et al., 2022). These two myokines may be involved in anabolic processes within skeletal muscle (Cornish et al., 2020). If these are elevated in younger individuals, it could explain how adaptations to skeletal muscle tissue may be enhanced more so by resistance exercise training in younger versus older muscle and partially explain the “anabolic resistance” experienced by older adults (Aragon et al., 2023). Of note, our cohort of older adults was excluded from participating if they were diagnosed with any health conditions (i.e., they were relatively healthy older adults); however, they did have higher pro‐inflammatory cytokines at baseline suggesting a chronic low‐grade inflammatory state.

A recent systematic review and meta‐analysis has demonstrated that exercise training in older adults, especially those with chronic disease, has a small to moderate effect on inflammatory biomarkers by producing a less inflammatory environment (Khalafi et al., 2023). Further research discovered that lifelong endurance training alleviated MCP‐1 and telomeric repeat‐containing RNA (TERRA) accumulation in skeletal muscle biopsies thus suggesting an alteration of one inflammatory biomarker and a biomarker that may delay cellular senescence, respectively (Balan et al., 2021). Although the results from Balan et al. (2021) are indicative of a preservation of essential inflammatory responses in older skeletal muscle, previous research from their laboratory indicates no effect of aerobic endurance training status on senescence with a reduction of skeletal muscle inflammation in older male individuals (Balan et al., 2020). Also, debate exists as to the best type of exercise to preserve skeletal muscle form and function with age but some research indicates that it may not matter when comparing endurance training to resistance exercise training (Bolotta et al., 2020). Here, the research found that there is a balanced response of the Akt–mTOR and AMPK pathways. This resulted in skeletal muscle maintenance with long‐term exercise training in older individuals participating in the different exercise types of exercise for 30 years or more; along with this finding, there was a reduced inflammatory profile within the long‐term exercisers (Bolotta et al., 2020). Again, these results suggest a crosstalk between skeletal muscle contraction and the innate immune system, particularly in those with chronic conditions that may elevate the inflammatory profile in humans. Certainly, the dysregulation noted in chronic conditions associated with skeletal muscle point to the idea that there is an inflammatory component which negatively influences skeletal muscle regeneration and, potentially, adaptation (Aragon et al., 2023; Degens & Korhonen, 2012; Fountain et al., 2023; Jo et al., 2012; Kunz & Lanza, 2023; Liang et al., 2022).

As delineated above in the younger adult section, M1 and M2 macrophages play a key role in muscle inflammation and regenerative processes. Some research has indicated that total macrophage number is not different between younger and older adults; however, older skeletal muscle M1 macrophage subtypes are lower at rest and following muscle damaging exercise compared to younger skeletal muscle (Ahmadi et al., 2022) suggesting alterations in how muscle inflammation may respond to exercise with age. This was supported by research indicating the M1 subtype of macrophage was lower in older adults compared to younger following muscle damaging exercise potentially negatively altering satellite cell function and muscle regenerative processes in older muscle (Sorensen et al., 2019). As reported above, research in humans has identified that M1 macrophages are decreased and M2 macrophages are increased in aged skeletal muscle (Cui et al., 2019). These authors also demonstrated that M2 macrophages colocalized with intermuscular adipose tissue and suggested that this may impact muscle metabolism adversely (Cui et al., 2019). Intermuscular adipose tissue exerts a pro‐inflammatory effect and thus may contribute to the increased amount of inflammation promoting skeletal muscle loss with age along with increasing amounts of adipose tissue (i.e., sarcopenic obesity) (Sheptulina et al., 2023). Also, evidence indicates M2 macrophages are associated with “regenerative inflammation” where pro‐regeneration, immunosuppression, and creation of an anti‐inflammatory milieu promote skeletal muscle tissue repair to clean up cellular debris and necrotic tissue and eventually restore tissue function (Caballero‐Sánchez et al., 2024). Furthermore, regulatory T cells (Treg) contribute to immunosuppression and promote M1 to M2 macrophage phenotypic shifts to allow for a coordinated response in skeletal muscle tissue (Caballero‐Sánchez et al., 2024). Interestingly, in a mouse model, Treg cells accumulate in injured skeletal muscle to a lesser degree in older versus younger animals which resulted in poor repair; however, introducing IL‐33 to the older animals restored the accumulation of Treg cells and enhanced skeletal muscle repair (Kuswanto et al., 2016).

In an excellent study that defined systemic differences between older adults with higher concentrations and lower concentrations of CRP, those individuals with high concentrations of CRP demonstrated that NF‐κB activation, proteosome activity, protein oxidation, and insulin dependent anabolic potential were all negatively altered in skeletal muscle when compared to those with lower levels of CRP (Draganidis et al., 2021). This study supports the contention that chronic low‐grade inflammation interferes with skeletal muscle in an adverse manner with age, thus once again demonstrating the crosstalk between skeletal muscle and the inflammatory response. Additionally, older adults have an increased proportion of M2 anti‐inflammatory macrophages following muscle damage compared to younger adults (Sorensen et al., 2019). This inhibited pro‐inflammatory response to muscle damaging exercise decreases satellite cell proliferation in older adults (Sorensen et al., 2019). Further defense of this is upheld by studies that found various pro‐inflammatory biomarkers are inversely associated with functional adaptation, skeletal muscle strength, and muscle quality in older individuals (Conte et al., 2020; Grosicki et al., 2020; Norheim et al., 2017; Rivas et al., 2012).

An in‐depth review of the topic of cytokine response by skeletal muscle to acute exercise has indicated there are many cell types located within skeletal muscle homogenate (the usual source of muscle tissue from human biopsy procedures) potentially influencing the cytokine secretion into the systemic circulation and skeletal muscle itself (Peake et al., 2015). Nonetheless, there is evidence demonstrating mRNA for various cytokines from human skeletal muscle biopsies; however, the protein production of these cytokines is likely upregulated in several cell types within the muscle in response to acute exercise and skeletal muscle contraction (Peake et al., 2015). This points out that more research is needed to completely understand if (and how) cytokines are being produced by skeletal muscle and communicating either with the muscle itself or other cells located within and outside the muscle.

The degree to which inflamm‐aging and immunosenescence are a result of the aging process or a result of reductions in physical activity levels with age is an interesting research question. In a series of two studies completed in males and females, the inflammatory response to acute resistance exercise was evaluated systemically and locally from skeletal muscle biopsies (Lavin et al., 2020a, 2020b). Here, the researchers found that older males who were lifelong exercisers responded similarly to the acute resistance exercise challenge when compared to younger males and had lower pro‐inflammatory biomarker levels as compared to older healthy non‐exercising males (Lavin et al., 2020a). However, in the older females that were lifelong exercisers, there was not a similar response as both resting and resistance exercise induced pro‐inflammatory reaction was not different between the exercisers and non‐exercisers in this female cohort (Lavin et al., 2020b). This dichotomy between biological sexes requires further investigation.

## 5 CONCLUSIONS

Exercising aging skeletal muscle will mitigate the loss of skeletal muscle mass and function. Our review highlights the current literature on the interplay between the immune system and skeletal muscle and the changes that accompany these systems with age. We highlight the important relationship between the immune system and skeletal muscle adaptation, the decreased M1 macrophage response to unaccustomed exercise in older adults and the negative implications this has on myogenic repair and regeneration, and the limited available research investigating the inflammatory profile of skeletal muscle in individuals with chronic disease. Future directions for research include (1) further identifying the potential sex related differences in the inflammatory response following exercise; (2) identifying possible mechanisms to target to improve muscle regeneration and repair after exercise in older adults; (3) investigating the inflammatory state of skeletal muscle in individuals with various chronic diseases; and (4) understanding more clearly what cell types produce cytokines as a result of acute exercise and skeletal muscle contraction. As more research is completed in this area, there will be an enhancement of the understanding surrounding how to lessen the loss of skeletal muscle mass and function with age and/or comorbid conditions affecting this tissue.

## FUNDING INFORMATION

No funding was received in the creation of this manuscript.

## CONFLICT OF INTEREST STATEMENT

SMC declares no conflicts of interest. DMC is affiliated with the Pan Am Clinic Foundation, which receives general education and research support from ConMed Linvatec, Ossur, Zimmer Biomet, and Arthrex.
